# Early diagnosis of breast and ovarian cancers by body fluids circulating tumor-derived exosomes

**DOI:** 10.1186/s12935-020-01276-x

**Published:** 2020-05-24

**Authors:** Leyla Norouzi-Barough, Amir Asgari Khosro Shahi, Farnoosh Mohebzadeh, Ladan Masoumi, Mohammad Reza Haddadi, Sadegh Shirian

**Affiliations:** 1 Department of Genetics and Molecular Biology, School of Medicine, Isfahan University of Medical Sciences, Isfahan, Iran; 2 Islamic Azad University, Kazerun Branch, Kazerun, Iran; 3 School of Medicine, Shiraz University of Medical Sciences, Shiraz, Iran; 4 Department of Neuroscience, School of Advanced Technologies in Medicine, Tehran University of Medical Sciences, Tehran, Iran; 5 Iranian National Center for Addiction Studies, Tehran University of Medical Sciences, Tehran, Iran; 6 Department of Pathology, School of Veterinary Medicine, Shahrekord University, Shahrekord, Iran; 7 Shiraz Molecular Pathology Research Center, Dr. Daneshbod Pathology Laboratory, Shiraz, Iran; 8 Shefa Neuroscience Research Center, Tehran, Iran

**Keywords:** Exosome, Breast cancer, Ovarian cancer, Biomarker, Diagnosis

## Abstract

Exosomes (EXs) are small extracellular vesicles, a size range of 40-100 nm in diameter, actively secreted by most eukaryotic cells into surrounding body fluids like blood, saliva, urine, bile, breast milk and etc. These endosomal-derived vesicles mediate cell–cell communication between various cell populations through transmitting different signaling molecules such as lipids, proteins, and nucleic acids, and participate in a wide range of physiological and pathological body processes. Tumor-derived EXs (TDEs) are vehicles for intercellular communications by transferring bioactive molecules; they deliver oncogenic molecules and contain different molecular cargoes compared to EXs delivered from normal cells, therefore, they can be used as non-invasive invaluable biomarkers for early diagnosis and prognosis of most cancers, including breast and ovarian cancers. Their presence and stability in different types of body fluids highlight them as a suitable diagnostic biomarker for distinguishing various cancer stages. In addition, EXs can predict the therapeutic efficacy of chemotherapy agents and drug resistance in cancer cells, as well as determine the risk of metastasis in different disease stages. In this study, the recent literature on the potential role of TDEs in the diagnosis and prognosis of ovarian and breast cancers is summarized, and then exosome isolation techniques including traditional and new approaches are briefly discussed.

## Introduction

Exosomes (EXs) were firstly reported to describe nano-sized exfoliated membrane vesicles with 5′-nucleotidase activity and physiologic function [1]. The best definition of EXs is extracellular vesicles which are released by most eukaryotic cells into the surrounding body fluids upon the fusion of multivesicular bodies (MVB) with the plasma membrane [2]. Their presence in different types of body fluids, such as blood, saliva, urine, bile, synovial fluid, breast milk, amniotic liquid, and seminal fluid, indicates their multiple key roles in intercellular communication via transferring both genomic and proteomic materials between cells and subsequently regulating physiological responses [3, 4]. Exosomes are secreted by a variety of normal cells, however, the evidence reveals that secretion of these nano-sized vesicles is more vigorous in pathological conditions, such as tumor cells, as they are detectable in sufficient quantities in specimens of patients with various types of cancer and in supernatant obtained from tumor cells in vitro [4, 5]. Due to the presence of tumor-specific antigens (TSA) in TDEs in addition to functional proteins, mRNAs, and miRNAs, they are considered as potential candidates for different clinical applications, including cancer diagnosis, prognosis, and treatment [6]. Since isolation of EXs from body fluids is relatively easy and considered as a noninvasive approach [7] and due to their specific active cargos, known as the “signature” of the donor cell, which mimic the cellular origin and its physiological and pathophysiological states [8] and considering their ability to provide a protective extracellular vesicle for transporting small RNAs against degradation of RNase, they can be used as worthy diagnostic biomarkers in various diseases, particularly some types of cancer, and can be considered as an ideal specimen for liquid biopsy for early detection of some types of cancers [9]. Here, we describe the application of TDEs for early diagnosis of breast and ovarian cancers.

### Breast and ovarian cancers

Globally, breast cancer (BC) is the most common type of malignity and the leading cause of cancer-related mortality among women. Annually, one in four new cancer cases belongs to female BC. In contrast, although ovarian cancer (OC) is rare, it is the leading cause of gynecological cancer-related mortality worldwide. In fact, ovarian carcinoma is one-tenth as common as BC but three times more lethal [10–12]. Because of the location of the ovaries within the pelvis, OC progresses asymptomatic in its early stages [13]. Therefore, the high mortality rate of OCs is in part due to late diagnosis and lack of proper screening. Accordingly, OC is known as a silent killer [14, 15]. Both BC and OC are considered as heterogeneous diseases, which are mainly the result of sporadic mutations, and just 5–10% of cases are attributed to a familial history [12, 16]. Due to their heterogeneity at both the molecular and clinical levels, current protocols for early detection of breast and OCs are either ineffective or expensive [16]. An appropriate screening method should preferably be efficient, non-invasive, easily executable and evaluable, safe, cost-effective and applicable to all individuals in the community. Thus, the identification of new biomarkers is needed for early diagnosis of BC and OC.

### Characteristics of exosomes and their composition

**Fig. 1:**
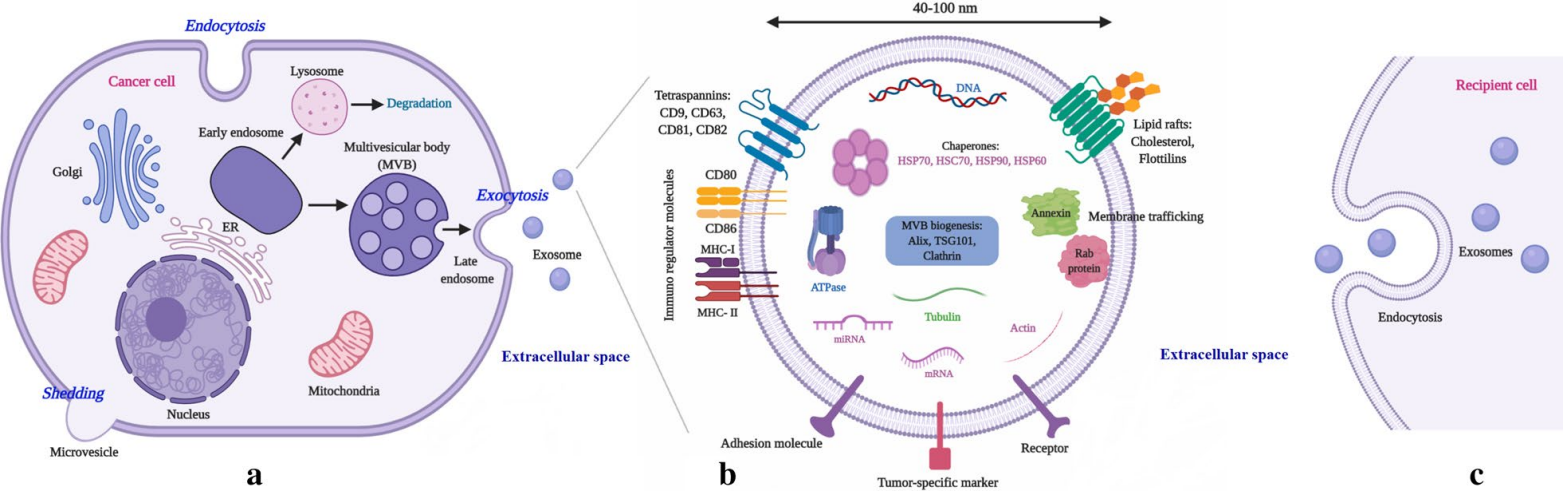
Biogenesis, structure, release, and uptake of exosomes. **a** Exosome biogenesis starts with inward budding of the plasma membrane (endocytosis) and the formation of early endosomes. Subsequently, incorporation of cytosolic proteins, lipids, and nucleic acids into the endosomes leads to the formation of multivesicular bodies (MVBs). Finally, MVBs fuse with the plasma membrane through RabGTPases pathway and exosomes are released into the extracellular space. **b** Exosomes are enriched by multiple families of proteins, including tetraspannins (CD9, CD63, CD81, CD82), heat shock proteins (Hsc70, Hsp 90, Hsp70, Hsp60), membrane trafficking proteins (Rabs, Annexins), proteins involved in MVBs biogenesis (Alix, TSG101, Clathrin), metabolic enzymes (GAPDH, ATPase, PGK1), cytoskeletal proteins (actin, vimentin, cofilin, tublin, talin), lipid rafts, such as cholesterol, flotillins, ceramides, sphingolipids, DNA, RNA species (mRNAs, miRNAs, lncRNAs) and tumor-specific markers. **c** Once exosomes are released into the extracellular space, they can interact with recipient cells via direct fusion, endocytosis or receptor-ligand interactions

Extracellular vesicles (EVs) include EXs and ectosomes (or shedding microvesicles; MVs) which are derived from endosomes and the plasma membrane, respectively. EVs are released by many types of cells and play a critical role in intercellular communication, presumably by serving as vehicles to transport membrane and cytosolic proteins, lipids, DNA (genomic DNA, mtDNA, cDNA) and RNA fragments (mRNAs, miRNAs, lncRNAs) between cells. The third type of EVs is apoptotic bodies (ABs), which are mainly secreted by dying or apoptotic cells and their role in intercellular communication is yet to be clarified [17–20]. EXs are commonly characterized by a size range of 40–100 nm in diameter [21], a density between 1.13 and 1.19 g/mL in a sucrose gradient, and a “saucer-like” or “cup-shaped” morphology when investigated by electron microscopy [22]. The size of EXs is partly influenced by their cellular source and the “cargo hold” of these nanoparticles is approximately 20–90 nm, therefore, it is estimated that the total burden per EXs is probably ≤ 100 proteins and ≤ 10,000 nucleotides [23]. Moreover, EXs are characterized by several families of proteins, including tetraspannins (CD82, CD81, CD63, CD9), heat shock proteins (Hsp 90, Hsp70, Hsp60, and Hsc70), membrane trafficking proteins (Rabs, Annexins), proteins involved in MVBs biogenesis (Alix, TSG101, Clathrin), metabolic enzymes (GAPDH, ATPase, PGK1), cytoskeletal proteins (actin, vimentin, cofilin, tublin, talin) as well as lipid rafts, such as cholesterol, flotillins, ceramides, and sphingolipids (Fig. 1) [24, 25]. CD63 and CD9 serve as the most commonly used EXs markers to distinguish them from other vesicles, such as MVs and ABs [26].

### Exosomes in cancer malignancy

The roles of TDEs in the development of cancer are due to their immunostimulatory or immunosuppressive properties and subsequently being involved in maintaining tumor cells survival, growth, and metastasis [22]. Tumor-secreted EXs are powerful intercellular signal mediators between tumor cells and their microenvironment, and their function seems to be dependent on their cargoes and surface biomolecule [27]. The immunological significance of EXs in cancer microenvironment is their potential to modulate the immune system in different ways in a host defense system. TDEs are capable of evading anti-tumor immune responses via different mechanisms [28]. For example, TDEs induce T cell apoptosis through carrying apoptosis-inducing ligands such as Fas ligand (FasL) and TNF-related apoptosis-inducing ligand (TRAIL), and galectin 9 [29]. On the other hand, they hamper IL-2-dependent activation of CD8^+^ T cell and natural killer (NK) cells mediated by TGF-β1 [29]. Moreover, exosomal programmed death-ligand 1 (PD-L1) can disrupt the activation and infiltration of T cells into the tumor microenvironment and promotes cancer metastasis and immune escape, but it is not clear whether the role of exosomal PD-L1 is tumor type-dependent or not [30]. Additionally, shedding of MICA/B ligands by tumor cells, as a component of TDEs, prevents NKG2D-mediated killing by CD8^+^ T cells, NK, and γδT cells [28]. Proteins and miRNAs carried by TDEs can modulate the expression of immune cells receptors like NKG2D and TLR4, thus suppress immune responses [29]. They also take part in inflammatory cell infiltration, angiogenesis, and transforming tumor cells into the more aggressive phenotype, including preparing the pre-metastatic microenvironment, via crosstalk with microenvironment cells, such as epithelial cells, macrophages, endothelial cells, and fibroblasts [31, 32]. Evidence shows that crosstalk between cancer cells-derived EXs and endothelial cells stimulate angiogenesis and metastasis in a hypoxic microenvironment [33]. Tumor exosomal miRNAs, as the main RNA content of EXs, participate in pre-metastatic niche formation and metastasis through interfering with the miRNA profile of target cells at distant organs. For instance, aggressive BC cells animate less-aggressive tumor cells to initiate metastasis and largely progress by EXs shedding and transfer functional RNAs [34]. Moreover, exosomal oncogenic proteins can promote the migration of tumor cells to the adjacent tissues [35]. As myoferlin present in EXs extracted from breast and pancreatic cancer cells contribute to cancer metastases with promoting exosomal biogenesis and transferring nucleic acids to adjacent cells [35]. Furthermore, EXs, as active transporter nucleic acids and oncogenic proteins to recipient cells, induce cancer drug resistance [36]. In general, EXs are key players in cancer development and progression by interaction with tumor microenvironment components including endothelial, fibroblastic, and immune cells and major tumor-related signaling pathways, such as cancer stemness, angiogenesis, hypoxia driven epithelial-to-mesenchymal transition (EMT), and metastasis [37, 38]. These interactions are needed for cancer growth and accelerate tumor drug resistance [38].

### Tumor exosomes as diagnostic biomarkers

Exosomal biomarkers have been found in different types of biological fluids, such as plasma, urine, blood, saliva, bile, bronchoalveolar lavage fluid, synovial fluid, cerebrospinal fluid, amniotic fluid, semen, breast milk, and malignant ascites [39]. TDEs present higher sensitivity and specificity compared to other body fluids biomarkers [39]. In addition, EXs have several other worthwhile advantages, including their extreme stability (under various conditions, such as cold-storage, freezing, and thawing for up to years), abundance (4000 trillion EXs in the blood of cancer patients versus 2000 trillion EXs in normal human blood), tumor-specificity, and the association of their content with tumor staging and treatment outcome [6, 32]. Since different types of tumors are characterized by tumor-specific miRNAs or proteins, exosomal cargos reflect the stage and degree of tumor progression [40]. Moreover, the presence of EXs in body fluids, such as blood and urine, highlights them as a non-invasive diagnostic and prognostic biomarker over invasive biopsies [6].

A strong correlation between mRNA and miRNA profiles of tumoral cells and EXs has been confirmed. Since the expression of miRNAs is dysregulated in most cancers, thus, different cancer types present different exosomal miRNAs signatures [7]. For example, microarray analysis revealed the overexpression of five miRNAs including hsa-miR-20a-5p, hsa-miR-24-3p, hsa-miR-106a-5p, hsa-miR-891a, and hsa-miR-1908 in EXs derived from patient sera with nasopharyngeal carcinoma compared to EXs from healthy controls [41]. Liu et al. (2017) reported that increased levels of exosomal miR-23b-3p, miR-10b-5p and miR-21-5p predict poor overall survival in non-small-cell lung cancer (NSCLC) patients [42]. Moreover, Sohn et al. (2015) reported that the expression levels of serum exosomal miR-18a, miR-221, miR-222, and miR-224 were obviously higher in hepatocellular carcinoma (HCC) patients than those with chronic hepatitis B (CHB) or liver cirrhosis (LC) [43].

On the other hand, EXs are involved in lipid-related pathologies; therefore, the lipid content of EXs may act as another molecular indicator for disease diagnosis and prognosis [8]. To date, based on the free web-based database, ExoCarta (www.exocarta.org), there are 41,860 protein, 4946 mRNA and 1116 lipid entries from 286 studies. Given the above, EXs and their specific cargos, as valuable sources of cell information, may better reflect the cellular processes during the development and progression of malignant tumors.

### Ovarian cancer-derived exosomal cargos and their potential role as biomarkers

The secretion of TDEs was first found in ascites and cyst fluids of OC patients [44]. Afterward, tumor EXs were found in other types of cancers, including melanoma, bladder, prostate, breast, colorectal, and brain cancers [45–47]. Among the mentioned cancers, OC patients are at high risk for mortality due to poor prognosis as well as late diagnosis at advanced stages [48]. Since there is no reliable method for early detection of OC, OC-derived EXs and their molecular cargos are considered as valuable biomarkers due to their large potential in early diagnosis and prognosis [49]. Im et al. (2014) have found that CD24 and epithelial cell surface antigen (EpCAM) are tumor-derived exosomal markers of OC cells using nano-plasmonic EXs (nPLEX) assay [50]. In another study, Zhao et al. (2016) have measured tumor-derived exosomal markers, CA-125, EpCAM, and CD24, in OC patients plasma using a microfluidic approach (ExoSearch) and reported that combination of these three exosomal markers can provide desirable diagnostic accuracy for early diagnosis of OC [51]. Taylor and Taylor (2008) could distinguish benign OC cases from patients with various stages of OC using microRNAs profiling EpCAM-positive EXs isolated from the peripheral blood. They have reported eight microRNAs as specific microRNAs defining different stages of OC from benign disease, including miR-141, miR-21, miR-200a, miR-200b, miR-200c, miR-214, miR-205 and miR-203 [52]. The results of a study that evaluated serum levels of exosomal miR-373, miR-200a, miR-200b, and miR-200c in a cohort of 163 patients with epithelial OC indicated that the concentrations of all four exosomal microRNAs in OC patients were higher compared to those in healthy control. Moreover, it has been shown that increased levels of miR-200c and miR-200b were associated with CA125 values and shorter overall survival [53]. Recently, Yoshimura et al. (2018) have reported that the expression level of exosomal miR-99a-5p is significantly up-regulated in sera of OC patients. Their results have indicated that this microRNA promotes cancer cell invasion by affecting neighboring human peritoneal mesothelial cells (HPMCs) via up-regulation of fibronectin and vitronectin. Therefore, serum miR-99a-5p may serve both as a potential detection biomarker and a target for inhibiting OC progression [54].

Szajnik et al. (2014) have revealed that plasma exosomal TGF-β1 and melanoma-associated antigen family (MAGE3/6) can be used to distinguish OC patients from those with benign tumors or healthy controls [55]. Li et al. (2009) have found that full-length membrane protein Claudin-4 can be shed from OC cells, therefore, claudin-containing EXs from the peripheral blood of ovarian carcinoma patients may be useful biomarkers for OC detection [56]. Moreover, Keller et al. (2009) have introduced L1CAM, CD24, ADAM10, and EMMPRIN as tumor exosomal biomarkers for early-stage diagnosis of OC [57]. In addition to peripheral blood circulating EXs, Zhou et al. (2015), using miRNAs profiling, have observed that urinary level of exosomal miR-30a-5p from ovarian serous adenocarcinoma patients was 3.3-fold higher than that in healthy women [58]. In a recent and innovative survey, researchers have analyzed circulating EXs and found diagnostic power of seven biomarkers, including HER2, EGFR, FRα, CA-125, EpCAM, CD24, and CD9 plus CD63, applying an integrated exosome profiling platform (ExoProfile chip) using only 10 µL plasma of OC patients. They have demonstrated that this biomarker panel obtained from molecular profiling of circulating EXs not only discriminated patient groups from benign subjects in a cohort of 20 samples but also successfully distinguished early and late-stage OC [59].

### BC-derived exosomal cargos and their potential role as biomarkers

BC is the most common type of malignity among women. The 5-year survival rate of BC patients who are diagnosed in early-stage (stage I), is 100%. Nevertheless, if it is diagnosed in late-stage (stage IV), the 5-year survival rate is reduced by almost 19% [10, 60]. Therefore, identification and development of new tools or sources for early diagnosis of cancer are urgently required. It has been shown that EXs are released from a variety of cancer cells, including BC [7, 61], therefore, breast TDEs can be used as worthwhile markers for early detection of BC. MicroRNAs-containing EXs may be considered as an ideal biomarker for early diagnosis of some diseases. Cancer-derived EXs are enriched by miRNAs compared to those released by non-tumourigenic cells [62]. On the other hand, the expression pattern of exosomal miRNAs may be correlated with the degree of tumor malignancy and prognosis [25]. In a cohort study, the levels of cell-free and exosomal miR-373, miR-372, and miR-101 in the serum of patients with different molecular subtypes of BC with healthy women have been compared. These researchers have found that the serum levels of cell-free miR-101 and miR-373 in the invasive BC were remarkably higher than BC benign subjects and healthy controls, but couldn’t discriminate between the BC subtypes. Moreover, they have reported that exosomal miR-373 can be considered as a blood-based biomarker for more aggressive tumors, including triple-negative and hormone receptor-negative BCs [63]. In addition, Zhou et al. (2014) have introduced serum-derived exosomal miR-105, which is a potent regulator of cell migration through direct interaction with the tight junction protein ZO-1, as a prognostic marker for prediction of metastatic progression in patients with BC. They have suggested that a combination of miR-105, with other blood-based miRNA and/or protein markers enhances the identification chance of BC patients with a high risk of metastasis [64]. In another study, using small RNA sequencing and qRT-PCR analysis of cellular and exosomal microRNAs from BC cell lines as well as mouse and human plasma samples, Hannafon et al. (2016) have reported that miR-1246 and miR-21 are selectively enriched in human BC EXs as well as in plasma of BC PDX mice [65]. Yoshikawa et al. (2018) have found that exosomal miR-223-3p levels are significantly higher in invasive ductal carcinoma (IDC) patients, the most common type of BC, compared to that in ductal carcinoma in situ (DCIS) BC patients and healthy controls. Their data indicated that plasma exosomal miR-223-3p levels were strongly associated with the malignancy of BC, therefore, EXs-encapsulated miR-223-3p might be a useful preoperative biomarker for the early diagnosis of invasive BC [66]. Interestingly, Gonzalez-Villasana et al. (2019) have isolated EXs containing miR-382, miR-155, and miR-145 from the serum of both BC patients and healthy individuals, but not in a selective manner in patients with BC. Nevertheless, they have detected a significantly high concentration of EXs in patients with different stages of BC compared to healthy individuals [67].

Rupp et al. (2011) have evaluated CD24 and EpCAM as exosomal markers from ovarian carcinoma ascites, serum, and pleural effusions of BC patients using anti-EpCAM or anti-CD24 magnetic beads. They have found that both EpCAM and CD24 are selectively present on cancer-derived EXs in ascites and pleural effusions, however, in BC patients EpCAM but not CD24 was absent from serum-derived EXs. These results propose that EpCAM and CD24 can be considered as suitable biomarkers for specific detection of cancer-derived EXs in ascites and pleural effusions. Moreover, they have suggested that CD24 could be an additional marker for the enrichment of TDEs which are present in the serum of breast and OC patients [68]. Wang et al. (2019) have investigated the potential application of the exosomal protein, CD82, which is one of the tetraspanin family members, in the diagnosis of BC patients of all stages and various histological subtypes. Their findings indicated that the expression level of CD82 was significantly down-regulated in BC tissue compared to that in healthy and benign breast disease tissues. Furthermore, a significant negative correlation was detected between expression levels of CD82 in tissues and EXs. This correlation is due to the redistribution of CD82 expression from tissues toward the blood with an effect on the development and metastasis of BC. Accordingly, exosomal CD82 expression levels may be useful to evaluate the metastatic potential of tumor cells and the prediction of cancer prognosis [69]. Proteomics techniques have revealed more candidate exosomal protein-based markers for early diagnosis of cancer. For instance, using liquid chromatography-tandem mass spectrometry to analyze circulating EVs isolated from plasma of BC patients (stages I and II) and healthy controls. Moon et al. (2016) have reported developmental endothelial locus-1 protein (Del-1) as a candidate exosomal protein biomarker, and mentioned that Del-1 may improve the identification of patients with early-stage BC in particular in combination with other diagnostic methods, such as MRI and clinicopathologic characteristics [70]. Furthermore, using mass spectrometry analyses, Melo et al. (2015) have found that glypican-1 (GPC1) is specifically enriched in cancer cell-derived EXs. They have reported that the level of GPC1-positive circulating EXs was increased in 75% of patients with BC compared to healthy individuals [71]. Although the diagnostic sensitivity of GPC1-positive EXs, as an individual biomarker for BC, is not sufficient, it may be improved in combination with other BC-associated exosomal markers [72]. The efficiency of applying the oncogenic protein Survivin, particularly Survivin-2B, from EXs extracted from the serum of patients with BC as a diagnostic and/or prognostic marker has been confirmed by Khan et al. (2014) [73]. A growing body of evidence suggests that tumor exosomal contents particularly miRNAs and proteins reflect the stage and subtype of some tumors. For instance, Stevic et al. (2018) have evaluated miRNA signatures in EXs derived from HER2-positive and triple-negative (TNBC) subtypes of BC and compered them with exosomal miRNAs in healthy women. They detected nine miRNAs including miR-27a, miR-27b, miR-335, miR-365, miR-376c, miR-382, miR-422a, miR-433, and miR-628 which differently expressed in EXs of either HER2-positive or TNBC patients compared with healthy individuals (Table 1) [74]. Moreover, Wang et al. (2019) have traced the lncRNA HOTAIR in circulatory EXs from BC patients and then investigated its pathological association with the status of the disease. Amongst diverse clinicopathological factors, a close correlation between the copy number of exosomal HOTAIR and tumoral expression of ErbB2 (HER2) has been observed, and this correlation was validated in BC cell lines [75]. Since molecular classification of BC into the different subtypes and stages is essential to select the best available treatment option and to develop new therapeutic strategies [76], identification of specific exosomal miRNAs as well as non-coding RNAs can provide novel insights into the EXs biology and its potential for monitoring the progression of disease [74]. In addition, the role of EXs in the prediction of chemotherapy-resistant BC has been investigated. As Yang et al. (2017) have reported for the first time, the GSTP1 content of EXs obtained from BC patients resistant to anthracycline/taxane-based chemotherapy was significantly higher than that in patients with partial or complete response to chemotherapy. Therefore, it has been suggested that GSTP1-containing EXs might be a diagnostic biomarker for chemo-resistant BC [77]. Besides, TDEs may carry cytotoxic agents with extracellular microenvironment origin and transfer them into other cells. Therefore, EXs may influence the potency and toxicity of chemotherapy agents. Barok et al. (2018) have reported a new mechanism of trastuzumab emtansine (T-DM1) that is mediated by EXs originated from HER2-positive cancer cells by binding of EXs to T-DM1 and contribute to the T-DM1 activity [78]. Taken together, these reports highlight the roles of tumor-derived circulating EXs as promising biomarkers for diagnosis and prognosis of BC, evaluation and monitoring of the therapeutic effect and outcome [25].

**Table 1:** Exosomal biomarkers in diagnosis and prognosis of breast and ovarian cancers

Exosomal marker	Cancer type	Clinical value	References
let-7, miR-200	OC SKOV-3 and OVCAR-3 cell lines	The exosomal let-7 miRNA expression was significantly greater in SKOV-3 (high invasive cell line) compared with OVCAR-3 (low invasive cell line) The expression of miR-200 family was only identified in OVCAR-3 cell-derived exosomes	[84]
miR-21, miR-23b, miR-29a	OC effusion supernatants	High expression all three exosomal microRNAs was associated with poor survival	[85]
miR-21, miR-141, miR-200a, miR-200b, miR-200c, miR-203, miR-205, miR-214	OC patients serum	Overexpression exosomal microRNAs in different stages of OC patients Exosomal microRNAs were significantly lower in benign OC patients and negative in control cases	[52]
miR-373, miR-200a, miR-200b, miR-200c	OC patients serum	Overexpression all four exosomal microRNAs in OC patients The levels of miR-200a, miR-200b, and miR-200c distinguished between malignant and benign OC The increased levels of miR-200c and miR-200b were associated with CA125 values and shorter overall survival	[53]
miR-99a-5p	OC patients serum	MiR-99a-5p significantly elevated in OC patients compared to benign patients and healthy individuals	[54]
miR-30a-5p	OC patients urine	High levels of miR-30a-5p in OC patients	[58]
EpCAM, CD24	OC patients ascite	High levels of EpCAM and CD24 in OC patients	[50]
EpCAM, CD24, CA-125	OC patients plasma	High levels of EpCAM, CD24, and CA-125 in OC patients	[51]
EpCAM, CD24, CA-125, HER2, EGFR, FRα, CD9, CD63	OC patients plasma	This panel distinguished early and late stage OC and discriminated patient groups from benign subjects	[59]
CD24, L1CAM, ADAM10, EMMPRIN	OC patients ascites	Malignant ascites-derived exosomes contained tumor progression related proteins CD24, L1CAM, ADAM10, and, EMMPRIN.	[57]
TGF-β1, MAGE3/6	OC patients plasma	TGF-β1, MAGE3/6 distinguished OC patients from benign group and healthy controls	[55]
Claudin-4	OC patients plasma	High levels of Claudin-4 in OC patients	[56]
CD24, EpCAM	OC patients ascite	EpCAM and CD24 were enriched in exosomes from ascites and pleural effusions	[68]
	BC patients pleural effusions and serum	EpCAM was absent from BC patients serum	
miR-373, miR-101	BC patients serum	High levels of miR-373 and miR-101 in BC patients compared to benign patients and healthy individuals Higher levels of miR-373 in more aggressive tumors (triple-negative and hormone receptor-negative BCs)	[63]
miR-105	BC patients serum	Overexpression of miR-105 in BC cells was associated with high risk of metastasis	[64]
miR-21, miR-1246	BC patients plasma	High levels of miR-21, miR-1246 in BC samples compared to healthy subjects	[65]
miR-223-3p	BC patients plasma	Higher levels of miR-223-3p in IDC group compared to DCIS patients and healthy controls	[66]
miR-27a, miR-27b, miR-335, miR-365, miR-376c, miR-382, miR-422a, miR-433, miR-628	BC patients plasma	miRNAs 27b, 335, 376c, 382, and 433 were upregulated in TNBC patients miRNAs 27a, 27b, 365, and 628 were upregulated in HER2-positive BC patients miR-422a was downregulated in HER2-positive BC patients	[74]
lncRNA HOTAIR	BC patients plasma	Positive correlation between the exosomal HOTAIR and HER2-positive BC patients	[75]
CD82	BC patients plasma	High levels of CD82 in BC exosomes is associated with high risk of metastasis	[69]
Del-1	BC patients plasma	Del-1 significantly elevated in BC patients compared to healthy controls	[70]
GPC1	BC patients serum	High levels of GPC1 in 75% of BC patients compared to healthy individuals	[71]
Survivin-2B	BC patients serum	Survivin significantly elevated in BC patients compared to healthy controls	[73]
GSTP1	BC patients serum	GSTP1 significantly elevated in chemo-resistant BC patients compared to chemo-sensitivite BC group	[77]

*OC* Ovarian cancer, *BC* Breast cancer, *IDC* Invasive ductal carcinoma, *DCIS* Ductal carcinoma in situ, *GPC1* glypican-1

### Conclusion and future perspective

Circulating TDEs containing TSA, and nucleic acids (especially, microRNAs) can be easily isolated using tumor markers and serve as non-invasive diagnostic and predictive biomarkers. Besides early detection, they can be used for prognosis and prediction therapeutic efficacy as well as developing metastatic disease based on their distinct molecular patterns between different stages of the disease and healthy control [25, 52]. These subcellular nano-particles are detectable in almost all the body fluids, however, in order to gain the best results considering the cancer type, selection of a suitable isolation protocol based on the downstream analysis, type, and volume of starting sample is critical [79]. Exosome isolation/purification protocols have been designed based on different protein markers, sizes, and density. However, few of these purification methods can efficiently isolate specific types of extracellular vesicles, including EXs [80]. Traditional isolation techniques include ultracentrifugation-based techniques, immune-affinity capture-based techniques, polymeric precipitation isolation, filtration, and liquid chromatography techniques [81, 82]. Differential centrifugation is the most widely and basic method for the separation of EXs from variety kinds of human samples. However, this technique has some restrictions such as being time-consuming, dependency on heavy equipments, losing a large number of EXs and reducing the yield and purity during the process [82]. Commercially available kits such as Invitrogen, 101Bio, Wako, and iZON may be considered as possible alternatives for quick and efficient separation of EXs from the small volume of samples [83]. To overcome some restrictions of traditional separation techniques, several novel exosome isolation methods have been recently developed. These comprise ultrafiltration separation, integrated double filtration microfluidic device, nanoplasmon-enhanced scattering (nPES), membrane-mediated exosome separation, and on-chip isolation of EXs [82]. Among all the aforementioned methods, density gradient centrifugation, chromatography (gel filtration), and nPES have been shown the most purity [82], but to eliminate the effect of normal cell EXs as well as large amount of samples and subsequently getting a high levels of pure exosome novel isolation and characterization approaches should be developed, which can be achieved via the cooperation of biology and bioengineering and the use of the Multi-Omics approaches [79]. Growing evidence suggests that EXs have the potential to be used as prognostic and early-stage diagnostic biomarkers of breast and ovarian cancers. Although, there is still a long way ahead of developing a reliable method with high-specificity for early detection of these malignancies, through the development of novel cancer-specific EXs-based screening tools, cancer prevention, and intervention strategies will be more efficient in the near future. Moreover, there is a significant need for performing large-scale clinical trials for further validation of the role of EXs as early diagnostic, predictive, and prognostic markers of breast and ovarian cancers and to evaluate their potential role in drug selection for personalized medicine.

## Data Availability

The datasets reviewed during the present study are available in the Pubmed repository.
